# Overexpression of *Arabidopsis* Nucleotide-Binding and Leucine-Rich Repeat Genes *RPS2* and *RPM1*(*D505V*) Confers Broad-Spectrum Disease Resistance in Rice

**DOI:** 10.3389/fpls.2019.00417

**Published:** 2019-04-05

**Authors:** Zhaowu Li, Jianzhong Huang, Zhangying Wang, Fen Meng, Siyuan Zhang, Xiaoqiu Wu, Zhihong Zhang, Zhiyong Gao

**Affiliations:** State Key Laboratory of Hybrid Rice, Key Laboratory for Research and Utilization of Heterosis in Indica Rice of Ministry of Agriculture, College of Life Sciences, Wuhan University, Wuhan, China

**Keywords:** *AtRPS2*, *AtRPM1*, rice, *Magnaporthe oryzae*, *Xanthomonas oryzae*, *Nilaparvata lugens*

## Abstract

The nucleotide-binding domain leucine-rich repeat (NLR) immune receptors play important roles in innate plant immunity. The activation of NLRs is specifically induced by their cognate effectors released from pathogens. Autoactive NLRs are expected to confer broad-spectrum resistance because they do not need cognate effectors to activate their immune responses. In this study, we demonstrated that the *NLR* genes *RPS2* and *RPM1*(*D505V*) from *Arabidopsis* were autoactive in *Oryza sativa* and conferred broad-spectrum resistance to fungal pathogen *Magnaporthe oryzae*, bacterial pathogen *Xanthomonas oryzae pv. oryzae* (*Xoo*), and pest brown planthopper (BPH, *Nilaparvata lugens* Stål). These results revealed that interfamily transfer of dicot NLRs to monocot species could be functional. The transgenic plants displayed early and strong induction of reactive oxygen species (ROS), callose deposition, and expression of defense-related genes after challenged with *M. oryzae*. The transcriptome analysis showed that the expressions of some defense-related genes were primed to adapt the transformed autoactive NLRs in the transgenic plants. This study indicates that autoactive NLRs are a promising resource for breeding crops with broad-spectrum resistance and provides new insights for engineering disease resistance.

## Introduction

Plants are constantly threatened by a wide range of pathogens in nature, and plants have evolved a complicated and multifaceted innate immune system to combat pathogen attacks. The first layer of the plant immune system is mediated with pattern recognition receptors (PRRs). PRRs perceive the pathogen-associated molecular patterns (PAMPs), the highly conserved molecular signatures across a wide range of pathogens, and activate the PAMP-triggered immunity (PTI). PTI contributes a basal immunity during pathogen infection (Jones and Dangl, 2006; Couto and Zipfel, 2016; Zipfel and Oldroyd, 2017). In most cases, PTI is sufficient to prevent pathogen invasion and disease development. Successful pathogens deploy virulent proteins which are named effectors to suppress PTI, and correspondingly, plants have evolved immune receptors to recognize the effectors and initiate rapid and strong defense responses, named effector-triggered immunity (ETI) (Bent and Mackey, 2007; Tsuda and Katagiri, 2010; Spoel and Dong, 2012). PTI is conserved in different plant species and acts as a basal defense, and ETI provides a race-specific resistance and amplifies PTI signaling to induce robust resistance responses. Therefore, PTI and ETI form a two-tier defense system to protect plants against various pathogens (Pieterse et al., 2014; Cui et al., 2015).

Most of the effector-recognizing immune receptors have a nucleotide-binding domain and a leucine-rich repeat domain, and these receptors are called NLR receptors. NLR receptors either directly or indirectly recognize effectors (Cesari et al., 2014). Only a few NLR receptors directly bind to their corresponding effectors. More NLRs recognize effectors *via* an indirect recognition. One indirect recognition model is the “guardee” strategy. RIN4 is guarded by NLR proteins RPM1 and RPS2 in *Arabidopsis* and is targeted by effectors AvrRpm1, AvrB, and AvrRpt2. When AvrB and AvrRpm1 are delivered into the plant cell, they induce the phosphorylation of RIN4, and the phosphorylated RIN4 activates RPM1 signaling (Mackey et al., 2003). RPS2 interacts with RIN4, and its activity is inhibited by RIN4. AvrRpt2 can cleave RIN4 to stimulate the activity of RPS2 (Mackey et al., 2002; Axtell and Staskawicz, 2003).

*NLR* genes are important resources for breeding resistant plants. Several *NLR* genes have been cloned and employed in practical applications, and obtained great economic and social benefits (Park et al., 2012; Wang et al., 2016). The genome of the dicotyledonous plant *Arabidopsis thaliana* contains about 150 *NLR* genes that encode CC-NB-LRR and TIR-NB-LRR two types of NLR proteins, and the genome of the monocotyledonous plant *Oryza sativa* contains about 480 *NLR* genes that encode only CC-NB-LRR proteins (Yang et al., 2006). Although the large amount of *NLR* genes has been found from genomes of different plant species, NLRs with known activation mechanisms are quite limited. Because NLRs specially recognize their cognate effectors to activate the immune responses, lacking the knowledge on their cognate effectors restricts the successful applications of those NLRs. Autoactive NLRs do not need effectors to activate their immune responses, and are potential effective resources for breeding broad-spectrum resistant plants. There are two ways to obtain autoactive NLRs. RPS2 is inhibited by RIN4 in *Arabidopsis*, it is autoactive in *rin4* knock-out mutant or transiently expressed in *Nicotiana benthamiana* (Day et al., 2005). Interfamily transfer of *NLR* genes such as *RPS2* to the plant species without their suppressive genes such as *RIN4* can obtain autoactive NLRs. NLRs have conserved domains to regulate the switch between inactive and active states. Mutations in certain domains can lead to autoactive NLRs. Activation of RPM1 requires phosphorylated RIN4, and it does not autoactive in *rin4* knock-out mutant. However, RPM1(D505V) is autoactive in *rin4* knock out mutant (Gao et al., 2011).

Rice (*Oryza sativa L.*) is an important staple food crop that feeds more than half of the world’s population. However, its production is threatened by many pathogens and insect pests (Ke et al., 2017). Rice blast disease, which is caused by the hemibiotroph ascomycete pathogen *M. oryzae*, is one of the most destructive diseases. *M. oryzae* infects rice stems, nodes, leaves, and panicles, resulting in 10–30% yield losses in many rice growing areas (Wu et al., 2015; Bundo and Coca, 2016; Wang B.H. et al., 2017). Bacterial blight is another devastating disease of rice, which is caused by *Xanthomonas oryzae pv. oryzae* (*Xoo*) (Gu et al., 2009). Brown planthopper (BPH) is a rice-specific herbivore and is one of the most destructive insects of rice (Cheng et al., 2013; Liu et al., 2015). *M. oryzae*, *Xoo*, and BPH cause massive yield decrease and are a huge threat to food security (Zhang and Wang, 2013; Jing et al., 2017). Therefore, it is an urgent issue to develop durable and broad-spectrum of rice cultivars against diseases and insects.

Some NLR proteins from both *Arabidopsis* and rice could induce programmed cell death (PCD) when they were transiently expressed in the leaves of *N*. *benthamiana* (Wulff et al., 2011). The results suggest that NLRs share conserved signal pathways among different species, and it is possible that NLR proteins from *Arabidopsis* can be used in rice to confer resistance. Interfamily transfer of *NLR* genes was seldom reported (Maekawa et al., 2012; Narusaka et al., 2013). We transformed the *Arabidopsis NLR* genes *AtRPS2* and *AtRPM1*(*D505V*) into the *Japonica* rice cultivar Nipponbare under the control of *Zea mays ubiquitin* promoter. The results showed that these transgenic rice plants increased resistance against fungal pathogen *M. oryzae*, bacterial pathogen *Xoo* and insect pest BPH. Evidence is provided to show that the dicot *NLR* genes can be functional and confer the broad-spectrum disease and pest resistance in monocot species. Our observations revealed that interfamily transfer *NLR* genes will broaden the resources for breeding multi-resistance crops.

## Materials and Methods

### Plant Materials and Growth Conditions

The rice used in this study was *Oryza sativa* ssp. *Japonica* var. Nipponbare. All of the rice plants were grown with soil in a greenhouse or in the experimental fields at Wuhan. The greenhouse was set with a cycle of 14 h light at 28°C/10 h dark at 25°C, and the relative humidity was 50–60%.

### Plasmid Construction and Rice Transformation

*AtRPS2* and *AtRPM1*(*D505V*) were cloned into a modified pCAMBIA1380 vector with an *ubiquitin* promoter. The constructs were transformed into the *Agrobacterium tumefaciens* strain EHA105. The *Agrobacterium*-mediated transformation and regeneration of transgenic plants were carried out as previously described (Chen et al., 2015). With hygromycin selection and segregation analysis, the single insertion stable transgenic plants and further progeny were used for subsequent experimental studies.

### Rice Blast Inoculations and Disease Resistance Evaluations

The *M. oryzae* isolates used in this study were grown on oatmeal agar for 1 week at 28°C and then transferred to blue light for 2 days to enhance sporulation. Fungal spores were suspended in 0.02% Tween-20 at a concentration of 5 × 10^5^/mL. Three to four-leaf stage rice plants were used for spray inoculation, and the inoculated plants were incubated under a dark moist condition (28°C, 99% relative humidity) for 24 h and then under a 12 h light/dark cycle with 80% humidity. Disease severity was evaluated at 7 days after inoculation by counting the lesion numbers, and measuring the lengths of the three largest lesions per leaf. For panicle blast inoculation, the upper-middle part of panicles was injected with 1 mL of conidial suspension at a concentration of 5 × 10^5^/mL, and panicle blast resistance was assessed at 14 days after inoculation by measuring the proportion of diseased panicles.

### Punch Inoculation and Root Infection Assay

For punch inoculation, detached rice leaves were lightly wounded with an ear punch, and 8 μL of spore suspension (5 × 10^5^/mL) was added to the wound sites. The inoculated leaves were incubated in a Petri dish contained sterile water with 0.1% 6-Benzylaminopurine (6-BA) to keep moisture. Lesions were measured at 7 days after inoculation. The fungal biomass in the infected rice leaf was quantified using the threshold cycle value (C_T_) of *M. oryzae Pot2* DNA against the C_T_ of rice genomic *actin* DNA (Park et al., 2008).

Root infection assays were carried out with moist vermiculite. The mycelial plugs were put into a 50 mL centrifuge tube filled with 25 cm of autoclaved moist vermiculite and covered with 3 mL of vermiculite. Rice seeds were surface- sterilized for 10 min and washed six times with autoclaved water. Three sterilized seeds were placed on the top of the inoculated vermiculite and covered with another 3 mL of vermiculite. The tubes were sealed with Parafilm to retain moisture and incubated for 3 weeks (22°C, 16 h light/8 h dark photoperiod) to examine lesions.

### Rice Sheath Infection Assay

To observe the infection process of *M. oryzae*, the spore suspensions of GC1-4 was inoculated on 6 cm-long leaf sheaths with the concentration of 5 × 10^5^/mL, and then the inoculated leaf sheaths were incubated under the same condition as spraying assay. The leaf sheaths were observed with a laser scanning confocal microscope (Leica SP8-X) at 24 and 48 hpi.

### *Xoo* Inoculation and Bacterial Blight Disease Assay

*Xoo* strain PXO99 was grown on PSA medium (300 g/L Potato, 5 g/L peptone, 15 g/L sucrose, 0.5 g/L Ca(NO_3_)_2_⋅4H_2_O, 2 g/L Na_2_HPO_4_⋅12H_2_O, 20 g/L agar, pH 6.8–7.0) for 2 days, then bacteria were suspended in sterile water and diluted to OD_600_ = 0.5. The three youngest fully expanded leaves of the 6 weeks old plants were inoculated with PXO99 by the leaf-clipping method. Lesion length and bacterial growth were measured at 14 dpi as previously described (Gu et al., 2009).

### BPH Resistance Evaluations

The BPH insects used for resistance evaluations were reared on the susceptible rice cultivar TN1. For honeydew excretion and BPH weight gain assay, BPH nymphs were weighed and released to a pre-weighed Parafilm sachet and tied to the rice leaf sheath. The honeydew of each Parafilm sachet and the insects were weighed after 48 h. The weight change of BPH was recorded as the weight gain of BPH, and the weight change of the sachet was recorded as the honeydew excretion.

To assess the survivals and durations of transgenic plants and Nipponbare after BPH infestation, 10 rice seedlings at 4 weeks old were infested with BPH at a rate of eight insects per seedling. The transgenic plants were examined and photographed when all Nipponbare seedlings were dead.

In host selection test, two 30 days old plants were grown in one plastic bucket. One plant is Nipponbare and the other is a transgenic plant. Twenty BPH nymphs were released in the bucket, and the number of BPH nymphs that settled on each plant was recorded at 3, 6, 12, 24, and 48 h after insect release.

### RNA Extraction and qRT-PCR Analysis

The total RNA was extracted using Trizol reagent (TaKaRa, Dalian, China), and cDNA synthesis was performed according to the manufacturer’s protocol (Thermo Fisher Scientific, Lithuania). qRT-PCR was carried out in a Bio-Rad CFX96 real-time system with SYBR green PCR kit. The *OsActin* gene was used as the internal control for data normalization. The primers used for qRT-PCR are listed in Supplementary Table S6.

### RNA-Seq Analysis

About 2 cm of leaf strips from 4 week-old Nipponbare and transgenic plants were collected, and total RNA of the samples was extracted by TRIzol reagent (TaKaRa, Dalian, China). The RNA quality was quantified using an Agilent 2100 bioanalyzer (Agilent, Santa Clara, CA, United States), and then sequenced at an Illumina HiSeqTM 2500 platform. The adaptors and low-quality sequences were removed from the raw data. Differentially expressed genes (DEGs) were identified using the criteria of fold change > 2 and *p* < 0.05.

### DAB Staining

Hydrogen peroxide was monitored by DAB staining as previously described (Shimono et al., 2012). Pieces of leaf sheaths (3 cm) were immersed in 1 mg/mL DAB solution (pH 3.8), followed by vacuum filtration for 10 min and incubation at room temperature for 10 h in the dark. Samples were bleached by boiling in ethanol for 15 min, and images were observed with a microscope (Olympus BX51, Tokyo, Japan).

### Callose Deposition Analysis

For examining callose deposition, rice seedlings were inoculated with *M. oryzae* GC1-4 for 24 and 48 h and washed in an ethanol:acetic acid (3:1 v/v) solution for 5 h. The seedlings were incubated in 70% ethanol for 2 h, in 50% ethanol for 2 h, and in water overnight. After rinsing three times with water, the seedlings were incubated with 10% NaOH for 1 h to make all tissues transparent. After being washed three times with water, the seedlings were stained with 0.01% aniline blue for 4 h (Hu et al., 2017). Representative images of callose deposition were observed using a laser scanning confocal microscope (Leica SP8-X) under the UV channel.

### Assessment of Agronomic Performance

The Nipponbare and transgenic plants were grown in a standard field in Wuhan, Hubei, China. When plants were harvested, the middle six plants in the central row of each plot were collected for agronomic trait measurement. The measured agronomic traits include: plant height, number of panicles per plant, filling rate per plant, 1000-grain weight (grams).

## Results

### Overexpression of *AtRPS2* and *AtRPM1*(*D505V*) Confers Resistance Against *M. oryzae* in Transgenic Rice

**FIGURE 1:**
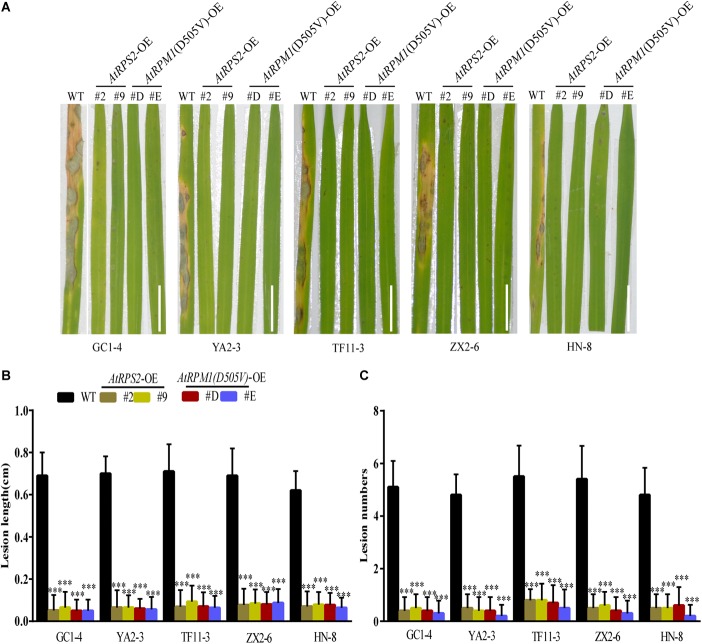
Overexpression of *AtRPS2* and *AtRPM1*(*D505V*) Confers Resistance Against *M. oryzae* in Transgenic Rice. **(A)** Representative disease symptoms on leaves of the *AtRPS2* and *AtRPM1*(*D505V*) transgenic plants and Nipponbare. Four-week-old plants were inoculated with different isolates of *M. oryzae*, and pictures were taken at 7 dpi. Scale bars = 2 cm. **(B,C)** Average lesion length and lesion numbers of *AtRPS2*, *AtRPM1*(*D505V*) transgenic plants and Nipponbare at 7 dpi inoculated with *M. oryzae* isolates. Data are shown as means ± SD (*n* = 20). Asterisks represent significant differences (one way ANOVA, ^∗∗∗^*P* < 0.001). Similar results were obtained in three independent experiments.

AtRPS2 is autoactive in plants without RIN4. AtRPM1 is not autoactive without RIN4 because the activation of *AtRPM1* needs the phosphorylated RIN4. However, AtRPM1(D505V) is autoactive without RIN4. Both *AtRPS2* and *AtRPM1*(*D505V*) can cause HR when transiently expressed in *N. benthamiana*. Since the autoactive NLRs usually result in broad-spectrum disease resistance, *AtRPS2* and *AtRPM1*(*D505V*) transgenic rice plants under the control of *ZmUbi* promoter were generated to determine the function of *AtRPS2* and *AtRPM1*(*D505V*) in rice. We obtained 51 independent *AtRPS2* transgenic T_1_ lines and 54 independent *AtRPM1*(*D505V*) lines. Among the T_1_ lines, 13 *AtRPS2* transgenic lines and 15 *RPM1*(*D505V*) transgenic lines grew so tiny that no seeds formed. Other lines showed various dwarf growth or normal growth. The transgenes were fused with HA tag, so the protein levels of *RPS2* and *RPM1(D505V)* in the transgenic lines could be determined. The detectable protein expressions of *RPS2* or *RPM1(D505V)* were found in the leaves of the dead T_1_ lines, while no detectable protein expressions in the survived T_1_ lines. The expression level of the genes in those transgenic lines were measured by qRT-PCR. The significant high lethal ratio and the dwarf growth of the transgenic plants indicate the autoactivation of *AtRPS2* and *AtRPM1(D505V)* in rice. Seven independent lines with stable single insertion and normal growth were selected for *AtRPS2* and *AtRPM1*(*D505V*) transgenic plants, respectively (Supplementary Tables S1, S2). Two lines per construct, *Ubi::AtRPS2*-2, -9 and *Ubi::AtRPM1*(*D505V*)-D, -E were chosen for experiments. They are lines with the highest expression level or the lowest expression level of transgenic genes based on qRT-PCR analyses (Supplementary Figures S1A,B). These transgenic lines and the transgenic background plant Nipponbare were used to evaluate the disease resistance against blast fungus. Five different *M. oryzae* isolates GC1-4, YA2-3, TF11-3, ZX2-6, and HN-8, which were collected from different cities or provinces of China and highly virulent to Nipponbare, were selected. Both *AtRPS2* and *AtRPM1*(*D505V*) transgenic plants showed obviously less disease severity compared to Nipponbare (Figure 1A). The lesion lengths and lesion numbers were dramatically reduced in the transgenic plants with these five isolates (Figure 1B,C). Similar results were obtained in detached leaf assays. The transgenic plants developed smaller lesions, and the lesion lengths on the transgenic plants were reduced to 40–50% compared to Nipponbare. Fungal DNA quantification indicated that the *AtRPS2* and *AtRPM1*(*D505V*) transgenic plants only harbored ∼35% *M. oryzae* compared to Nipponbare (Supplementary Figure S2).

**FIGURE 2:**
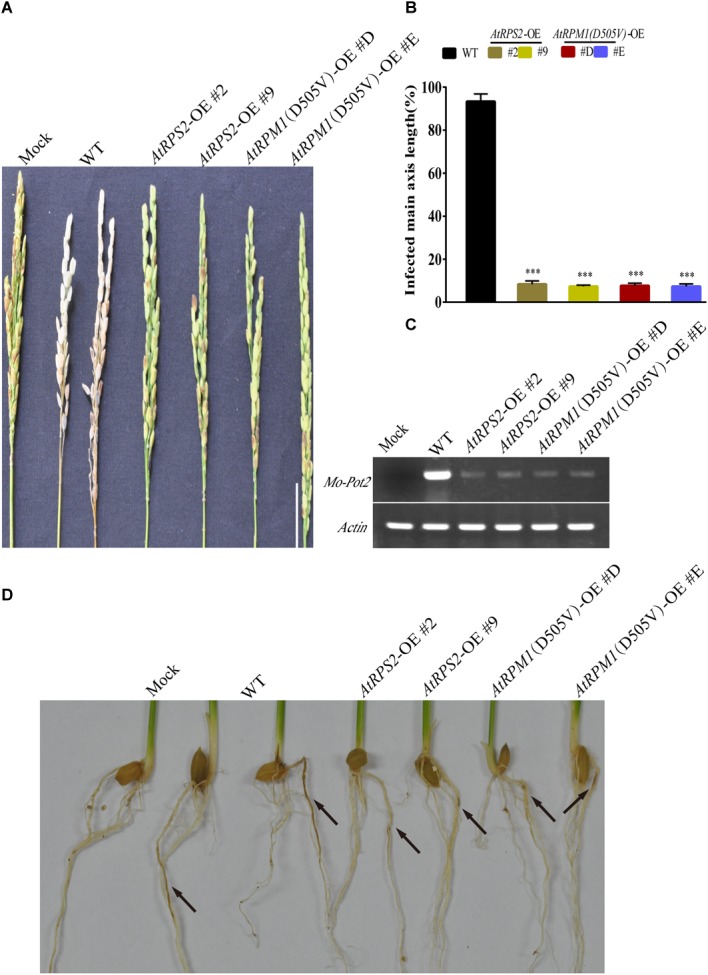
Panicle Blast and Root Blast Infection Assays of the *AtRPS2* and *AtRPM1*(*D505V*) Transgenic Plants and Nipponbare. **(A)** Disease phenotypes of the transgenic plants and Nipponbare after inoculation with *M. oryzae* isolate GC1-4 at the heading stage. Scale bars = 4 cm. **(B)** Lengths of the diseased main axis in the *AtRPS2*, *AtRPM1*(*D505V*) transgenic plants and Nipponbare after panicle blast infection. Data are shown as means ± SD (*n* = 6). Asterisks represent significant differences (one way ANOVA, ^∗∗∗^*P* < 0.001). **(C)** The fungal growth in infected panicles was measured by semiquantitative RT-PCR. **(D)** Disease symptoms on the roots of the transgenic plants and Nipponbare after *M. oryzae* isolate GC1-4 infection. Seedlings were examined for disease symptoms after 3 weeks incubation. Black arrows indicate the lesions of roots. Similar results were obtained in three independent experiments.

The panicle blast resistance was tested with the transgenic plants. The whitish dead panicles were observed in Nipponbare at 14 days post-inoculation (dpi), while none of the *AtRPS2* and *AtRPM1*(*D505V*) transgenic plants exhibited disease symptoms (Figure 2A,B). The semi-quantitative RT-PCR assay indicated that the fungal biomass in Nipponbare was much higher than those in the transgenic plants (Figure 2C). In the root infection assay, the roots of Nipponbare showed obvious necrotic symptoms, while the roots of the transgenic plants displayed less necrotic symptoms (Figure 2D). These results suggest that *AtRPS2* and *AtRPM1*(*D505V*) induce strong blast resistance in leaves, panicles, and roots of rice.

**FIGURE 3:**
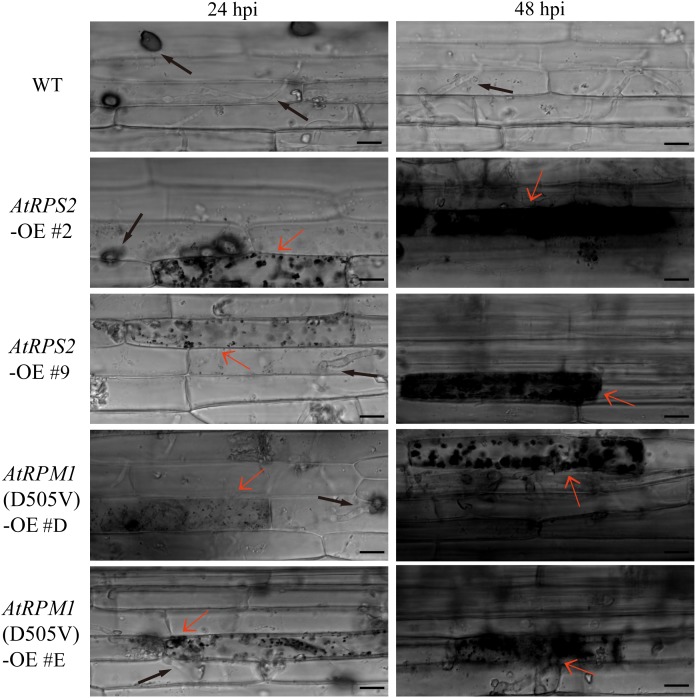
*AtRPS2* and *AtRPM1*(*D505V*) Restrict *M. oryzae* Invasion in Transgenic Plants. Representative microscopic images showing the growth of the *M*. *oryzae* isolate GC1-4 at 24 and 48 hpi on the sheath cells of the transgenic plants and Nipponbare. Black arrows indicate the appressorium and invasive hyphae, and red arrows indicate the plant cell death. Scale bars = 20 μm.

Leaf sheaths were inoculated with conidial suspensions of *M. oryzae* isolate GC1-4 to microscopically monitor the growth of the pathogen in plants. High levels of invasive hyphae and extensions to neighboring cells were observed in Nipponbare at 24 h post-inoculation (hpi), while only a small percentage of spores developed invasive hyphae and some plant cells displayed cell death in the transgenic plants. Almost all the penetration sites showed high levels of invasive hyphae in Nipponbare at 48 hpi, while the infection sites generated severe HR death response in the transgenic plants (Figure 3).

### Overexpression of *AtRPS2* and *AtRPM1*(*D505V*) Confers Enhanced Defense Responses in Transgenic Rice

**FIGURE 4:**
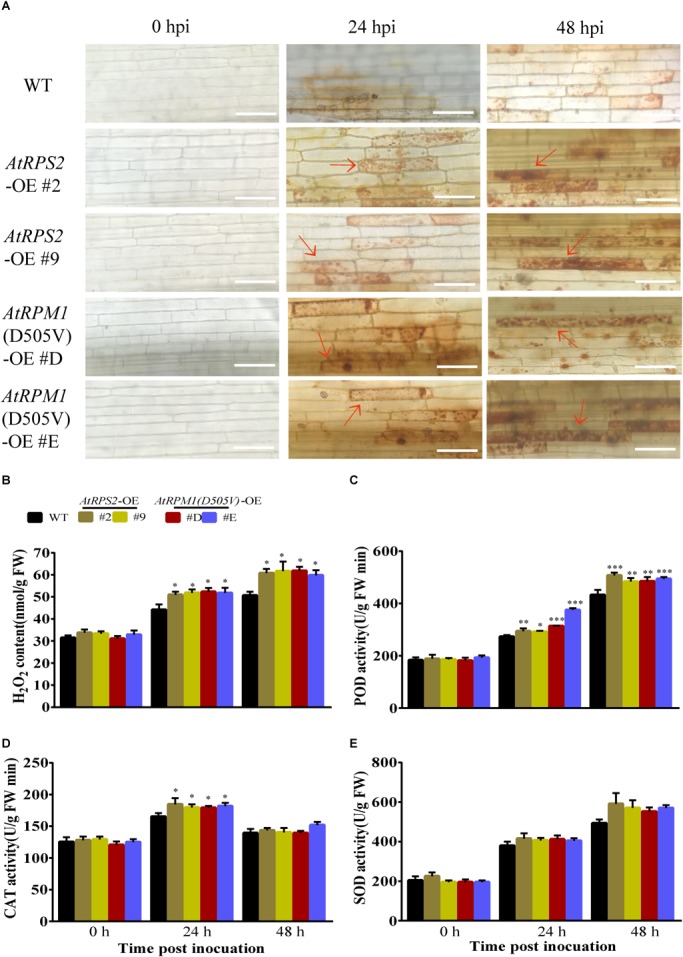
*AtRPS2* and *AtRPM1*(*D505V*) Trigger Strong ROS Induction During *M. oryzae* Infection. **(A)** DAB staining of the transgenic plants and Nipponbare at 24 and 48 hpi. There were many tawny shading cells in the transgenic plants, but in Nipponbare, the infected cells mostly exhibited light yellow color. Red arrows indicate dead plant cells. Scale bars = 50 μm. **(B)** H_2_O_2_ contents after inoculation with *M. oryzae* isolate GC1-4 in Nipponbare and transgenic plants. FW, fresh weight. **(C–E)** The enzymatic activities of POD, CAT, and SOD were assayed after *M. oryzae* inoculation. Data are shown as means ± SD (*n* = 3). Asterisks denote significant differences (one way ANOVA, ^∗^*P* < 0.05, ^∗∗^*P* < 0.01, ^∗∗∗^*P* < 0.001). Similar results were obtained in three independent experiments.

Reactive oxygen species (ROS), such as H_2_O_2_, are often correlated with plant defense responses (Liu et al., 2016; Yuan et al., 2017). In this study, 3,3-diamino-bezidine (DAB) was used to detect H_2_O_2_ in the leaves of the plants. While the leaves of Nipponbare and the transgenic plants had no H_2_O_2_ staining without the inoculation of *M. oryzae*, the transgenic plants generated much higher amounts of H_2_O_2_ than Nipponbare at 24 and 48 hpi (Figure 4A,B). We analyzed the activities of peroxidases (POD), catalases (CAT), and superoxide dismutases (SOD) in the plants. Before pathogen inoculation, the activities of these enzymes had no obvious differences between Nipponbare and the transgenic plants. Inoculation of *M. oryzae* increased the activities of the three enzymes, and the transgenic plants had higher POD and CAT activities than Nipponbare at 24 hpi (Figure 4C,D). The SOD activities in transgenic plants were higher than Nipponbare but did not display significant differences after *M. oryzae* inoculation (Figure 4E). These results indicate that expression of *AtRPS2* and *AtRPM1*(*D505V*) in rice can increase H_2_O_2_ accumulation corresponding to the inoculation of *M. oryzae*.

**FIGURE 5:**
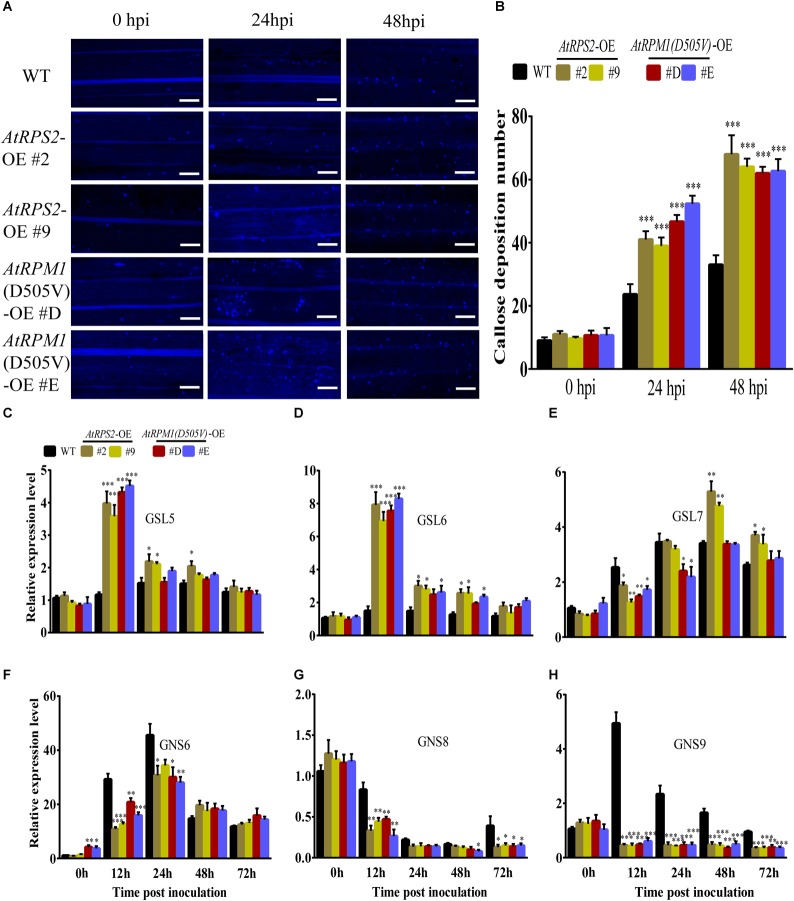
*AtRPS2* and *AtRPM1*(*D505V*) Promote Callose Deposition During *M. oryzae* Infection. **(A)** Callose depositions were obviously increased in the transgenic plant cells compared with the Nipponbare cells at 24 and 48 hpi. Scale bars = 50 μm. **(B)** Mean values of the callose deposition number. Data are shown as means ± SD (*n* = 5). **(C–H)** Expression analysis of callose synthase genes and callose hydrolase-encoding genes upon *M*. *oryzae* infection. The *OsActin* gene was used as an internal control. Gene expression levels were normalized to 0 h of Nipponbare. Data are shown as means ± SD (*n* = 3). Asterisks denote significant differences (one way ANOVA, ^∗^*P* < 0.05, ^∗∗^*P* < 0.01, ^∗∗∗^*P* < 0.001). Similar results were obtained in three independent experiments.

Callose deposition is another important defense response and can prevent pathogens from penetrating into the plant cells (Shangguan et al., 2018). The callose depositions on the leaves of rice were stained with aniline blue at 0, 24, and 48 hpi, and the transgenic plants displayed more callose depositions than Nipponbare (Figure 5A,B). We determined the expression patterns of callose biosynthesis genes, *OsGSL5*, *OsGSL6*, and *OsGSL7*. *M. oryzae* induced the highest expressions of *OsGSL5* and *OsGSL6* in *AtRPS2* and *AtRPM1* transgenic plants at 12 hpi, but the two genes did not have significant expression changes in Nipponbare (Figure 5C,D). *OsGSL7* displayed similar expression patterns in all transgenic plants and Nipponbare (Figure 5E). We also determined the expression patterns of callose hydrolase genes, *OsGNS6*, *OsGNS8*, and *OsGNS9*. *OsGNS6* was induced by the inoculation of *M. oryzae* in the transgenic plants and Nipponbare, but the transgenic plants had lower expression levels than Nipponbare at 12 and 24 hpi (Figure 5F). *OsGNS8* displayed similar expression patterns in the transgenic plants and Nipponbare, but the transgenic plants significantly reduced the gene expression at 12 hpi (Figure 5G). *OsGNS9* displayed different expression patterns between the transgenic plants and Nipponbare. The expression of *OsGNS9* was constantly downregulated in the transgenic plants after *M. oryzae* inoculation, while the expression of *OsGNS9* was dramatically upregulated in Nipponbare at 12 and 24 hpi (Figure 5H). Therefore, the early induced *OsGSL5* and *OsGSL6*, as well as the constantly suppressed *OsGNS9* promote the synthesis of callose and reduce the hydrolysis of the callose as well, resulting in quick and strong callose depositions in the transgenic plants.

### The *AtRPS2* and *AtRPM1*(*D505V*) Transgenic Plants Are Primed for Defense Responses

**FIGURE 6:**
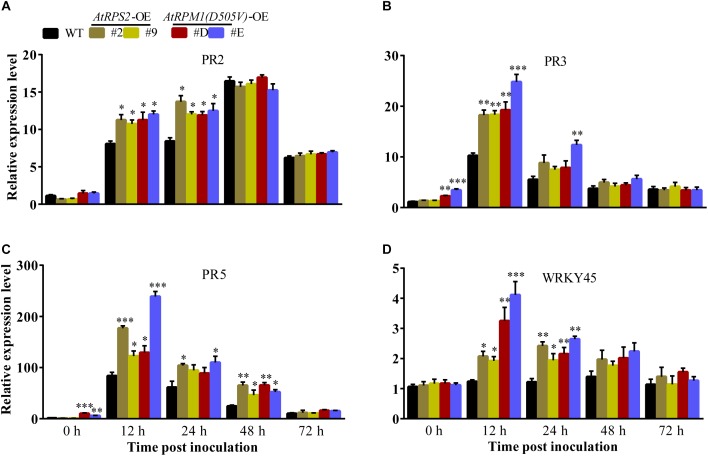
Overexpression of *AtRPS2* and *AtRPM1*(*D505V*) Trigger the Expression of Defense Responsive Genes in Transgenic Rice. **(A–D)** *AtRPS*2 and *AtRPM1*(*D505V*) promoted the expression of rice defense-responsive genes after inoculating *M. oryzae* isolate GC1-4; the *OsActin* gene was used as an internal control. Gene expression levels were normalized to 0 h of Nipponbare. Data are shown as means ± SD (*n* = 3). Asterisks denote significant differences (one way ANOVA, ^∗^*P* < 0.05, ^∗∗^*P* < 0.01, ^∗∗∗^*P* < 0.001). Similar results were obtained in three independent experiments.

The transgenic plants did not display constitutively higher contents of H_2_O_2_ and callose deposition, suggesting that *AtRPS2* and *AtRPM1*(*D505V*) did not constitutively activate the plant immunity in rice. The early and strong inductions of H_2_O_2_ and callose deposition indicate that the transgenic plants are primed for defense responses. We analyzed the expression of some defense-responsive genes in the transgenic plants and Nipponbare. Upon *M. oryzae* inoculation, the transgenic plants displayed higher expression levels of *PR2* than Nipponbare at 12 and 24 hpi (Figure 6A). *PR3* and *PR5* were induced to higher levels in the transgenic plants than in Nipponbare at 12 hpi, and the two genes displayed obviously constitutive expression in the *AtRPM1*(*D505V*) transgenic plants (Figure 6B,C). The expression level of *WRKY45* in Nipponbare was not induced upon *M. oryzae* challenge, while its expression was induced to higher levels in the transgenic plants at 12 and 24 hpi (Figure 6D). These results suggest that the expression of *AtRPS2* and *AtRPM1*(*D505V*) leads to the quick and strong induction of the defense- related genes upon pathogen infection.

We compared the global gene expressions between the transgenic plants and Nipponbare without pathogen inoculation. Compared to Nipponbare, the *AtRPS2* transgenic plants and the *AtRPM1*(*D505V*) transgenic plants had 106 and 245 upregulated genes, and 86 and 148 downregulated genes, respectively (Supplementary Tables S3, S4). We analyzed these DEGs using the gene ontology (GO) analyses. In *AtRPS2* transgenic plants, the expression of genes involved in “metabolic process,” “defense response,” and “zeatin activity” showed significant enrichment, and in *AtRPM1*(*D505V*) transgenic plants, the expression of genes involved in “metal ion homeostasis,” “defense response,” and “metal ion binding” showed significant enrichment (Supplementary Figures S3, S4). Because *AtRPS2* and *AtRPM1*(*D505V*) transgenic plants displayed similar disease resistance, we selected the commonly regulated genes in both transgenic plants. 97 genes were commonly regulated in both transgenic plants, and 50 genes had annotations (Supplementary Table S5). Most of the genes are defense-related genes, including eight putative *NLR* genes and four *receptor-like kinase* genes. These results indicate that expression of *AtRPS2* and *AtRPM1*(*D505V*) causes the plant to be primed for disease resistance.

### Overexpression of *AtRPS2* and *AtRPM1*(*D505V*) Confers Resistance to Bacterial Pathogen *Xoo* and BPH in Transgenic Rice

**FIGURE 7:**
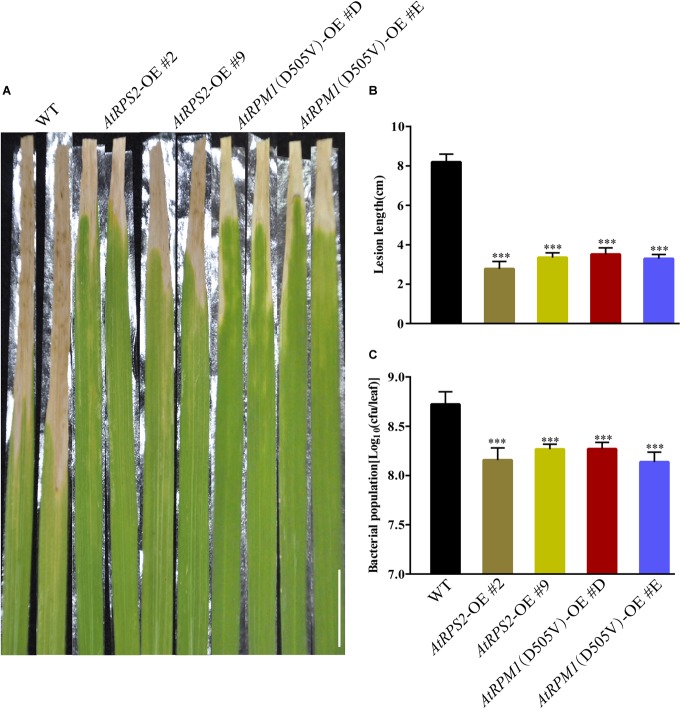
*AtRPS2* and *AtRPM1*(*D505V*) Confer Resistance to Bacterial Pathogen *Xoo* in Transgenic Plants. **(A)** Representative disease phenotypes on leaves of the *AtRPS2*, *AtRPM1*(*D505V*) transgenic plants and Nipponbare plants at 14 dpi with *Xoo* strain PXO99. Scale bars = 2 cm. **(B,C)** Lesion lengths and bacterial growth in the inoculated leaves of the transgenic plants and Nipponbare at 14 dpi. Data are shown as means ± SD (*n* = 15). Asterisks denote significant differences (one way ANOVA, ^∗∗∗^*P* < 0.001). Similar results were obtained in three independent experiments.

Because *AtRPS2* and *AtRPM1*(*D505V*) enhanced resistance to blast disease and could trigger stronger defense responses, we tested whether the *AtRPS2* and *AtRPM1*(*D505V*) transgenic plants showed enhanced resistance to bacterial blight disease, which was caused by *Xoo*. The transgenic plants showed remarkably shorter lesion lengths caused by *Xoo* strain PXO99 compared to Nipponbare at 14 dpi (Figure 7A). The average lesion length of the transgenic plants was 3.5 cm, while the average lesion length of Nipponbare was 8.2 cm (Figure 7B). Similarly, the bacterial growth rates in the transgenic plants were significantly less compared to Nipponbare (Figure 7C). These data suggest that overexpression of *AtRPS2* and *AtRPM1*(*D505V*) in rice enhanced resistance to bacterial pathogen *Xoo*.

**FIGURE 8:**
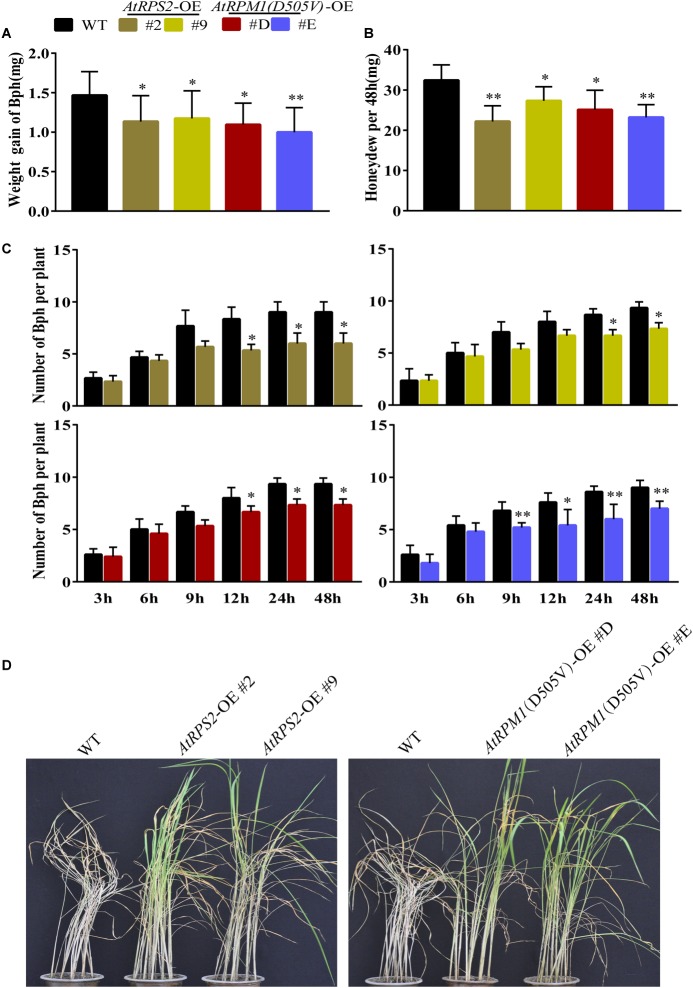
*AtRPS2* and *AtRPM1*(*D505V*) Confer Resistance to BPH in Transgenic Plants. **(A,B)** Weight gain and Honeydew excretion of BPH on transgenic plants and Nipponbare after feeding 2 days. Data were shown as means ± SD (*n* = 20). **(C)** Host choice test of BPH on transgenic plants and Nipponbare plants. Data are shown as means ± SD (*n* = 10). Asterisks denote significant differences (one way ANOVA, ^∗^*P* < 0.05, ^∗∗^*P* < 0.01). Similar results were obtained in three independent experiments. **(D)** Representative images of the transgenic plants and Nipponbare plants after BPH infestation. Note that the Nipponbare seedlings were dead thoroughly, but the transgenic plants still survived.

We also determined whether the transgenic plants developed enhanced resistance to the phloem-sucking insect BPH. We measured the weight gains of BPH and the honeydew excretions of the insects after feeding on the plants 2 days. The insects gained less weight and produced less honeydew on the transgenic plants (Figure 8A,B). Host choice test showed that Nipponbare was more attractive to BPH after 12–48 h release (Figure 8C). Furthermore, the transgenic plants survived from the BPH infection when all the Nipponbare plants had been killed by the insects (Figure 8D). BPH weight change and honeydew excretion reflect the antibiosis of the plants to the insects, and the host choice reflects the antixenosis of the plants to the insects. The results suggest that *AtRPS2* and *AtRPM1*(*D505V*) also confer the enhanced resistance to BPH.

### *AtRPS2* and *AtRPM1*(*D505V*) Transgenic Plants Exhibit Fitness Costs

**FIGURE 9:**
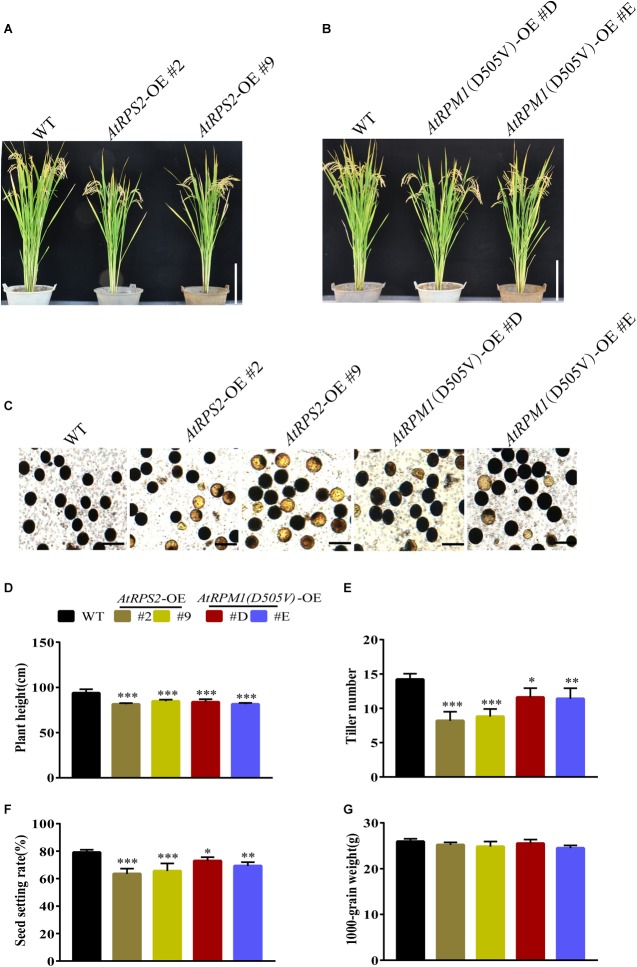
Phenotypes and agronomic traits of the *AtRPS2*, *AtRPM1*(*D505V*) transgenic plants and Nipponbare plants. **(A,B)** The morphological phenotypes of AtRPS2, AtRPM1(D505V) transgenic plants and Nipponbare. **(C)** Comparison of pollen fertility between the transgenic plants and Nipponbare plants. The heavily stained pollen is fertile and the lightly stained pollen is infertile. **(D–G)** Plant height (cm), tiller numbers per plant, seed setting rate, and 1000-grain weight. Data are shown as means ± SD (*n* = 6). Asterisks represent significant differences (one way ANOVA, ^∗^*P* < 0.05, ^∗∗^*P* < 0.01, ^∗∗∗^*P* < 0.001).w9

The constitutive expression of *AtRPS2* or *AtRPM1*(*D505V*) conferred the enhanced disease resistance, but it also brought in the fitness cost. We grew the transgenic plants and Nipponbare in the experimental fields to examine the agronomic traits. At mature stage, the *AtRPS2* and *AtRPM1*(*D505V*) transgenic plants were shorter than Nipponbare (Figures 9A,B). In addition, the transgenic plants possessed more infertile pollen (Figure 9C). The yield agronomic traits including plant height, tiller numbers per plant, seed setting rates and 1000-grain weights were measured. There was no difference between the transgenic plants and Nipponbare in 1000-grain weight, while the plant height, tiller numbers per plant, seed setting rates were significantly lower in the transgenic plants (Figures 9D–G).

## Discussion

The transfer of the PRR genes and resistance-related genes from *Arabidopsis* to other plants has been proposed as a strategy for achieving broad-spectrum resistance. The *Arabidopsis* PRR EF-Tu receptor (EFR) recognizes the bacterial elongation factor Tu and elf18. Expression of *AtEFR* in tomatoes and *Nicotiana benthamiana* conferred resistance to various bacterial pathogens (Lacombe et al., 2010), and expression of *AtEFR* in rice and wheat enhanced resistance to bacterial pathogen *Xoo* and *Pseudomonas syringae pv. Oryzae*, respectively (Lu et al., 2015; Schoonbeek et al., 2015). The expression of *AtRPW8.1* in rice conferred rice blast and bacterial blight resistance (Li et al., 2018). NPR1 is a positive regulator of the systemic acquired resistance (SAR) in *Arabidopsis* (Fu and Dong, 2013). Expression of *AtNPR1* in rice enhanced resistance to the fungal pathogen *Rhizoctonia solani* and bacterial pathogen *Xoo* (Fitzgerald et al., 2004; Molla et al., 2016). Therefore the successful usage of *Arabidopsis* PRRs and resistance-related genes in other species indicates that the immunity pathways among different plants are relatively conserved.

Different from previous research, we transformed the *Arabidopsis* autoactive *NLR* genes *AtRPS2* and *AtRPM1*(*D505V*) into the rice cultivar Nipponbare. The autoactivity of the two genes in rice can be proven by the tiny growth appearing on the transgenic plants with high expressions of the two genes. Because *Agrobacterium*-mediated gene transfer randomly integrates the transgenic gene to the rice genome, some transgenic plants with low expression of the two genes grew normally. The cognate effector of *AtRPS2* is AvrRpt2 from *Erwinia amylovora*, and the cognate effectors of *AtRPM1* are AvrRpm1 and AvrB from *Pseudomonas syringae*. The two NLRs should specially fight against pathogens contained the cognate effectors, however, the autoactive *AtRPS2* and *AtRPM1*(*D505V*), which did not need their cognate effectors to activate their function in rice, conferred enhanced resistance to fungal pathogen *M. oryzae*, bacterial pathogen *Xoo*, and insect pest BPH (Figure 1, 7, 8). In addition, the low expression transgenic lines display strong resistance, which means that we could choose the low expression autoactive transgenic lines to minimize fitness cost. Our study suggests that NLRs share conserved signaling pathways among different species, and autoactive NLRs confer plants broad-spectrum resistances.

Because *AtRPS2* and *AtRPM1*(*D505V*) were constitutively expressed in rice, we previously expected the transgenic plants to have constitutive defense responses, such as high H_2_O_2_ content and callose depositions. However, both *AtRPS2* and *AtRPM1*(*D505V*) transgenic plants did not display detectable higher H_2_O_2_ content or more callose depositions compared to the wild type plant Nipponbare (Figure 4, 5). The early and strong defense responses were induced in transgenic plants by the inoculation of *M. oryzae*, which ultimately suppressed the spread of invasive hyphae.

Compared with the global gene expressions in Nipponbare, two putative receptor-like kinase genes *OS11G0274100* (similar to stem rust resistance protein) and *OS11G0470500* were upregulated 8- and 3-fold, respectively, in the *AtRPM1*(*D505V*) transgenic plant, and two putative receptor-like kinase genes *OS10G0162836* (Wall-associated receptor kinase 1, WAK1) and *OS11G0226201* (similar to HSL2) were downregulated 4- and 33-fold, respectively (Supplementary Table S5). WAK1 is a candidate receptor of oligogalacturonides (OGs), components released from the plant cell wall (Brutus et al., 2010). HSL2 reduces leaf pectin content and leaf robustness, and HSL2 knockdown lines increase *Arabidopsis* resistance to *Pst* DC3000 (Wang X. et al., 2017). These data suggest that the cell wall structure and some PRRs associated with cell wall or on the plasma membrane have constitutively changed in the transgenic plants. Both *AtRPS2* and *AtRPM1*(*D505V*) transgenic plants displayed the same resistance, and it was consistent with the data that they share many commonly regulated genes.

The fitness costs resulting from autoactive NLRs hurdle the practical application of such NLRs. We used the *Zea mays ubiquitin* promoter to control the gene expression of *AtRPS2* or *AtRPM1*(*D505V*) in rice. The constitutive and high expression promoter was used to produce sufficient protein levels in rice, thus determining the autoactivity of the NLRs in the transgenic plants and subsequent resistance analysis. We obtained a series of transgenic plants with phenotypes ranging from dwarf plants to wild-type-like plants. The random insertions of the transfer genes into the genome can produce transgenic plants with different expression levels, allowing the choice of transgenic plants with less fitness costs while maintaining broad-spectrum resistance. The induction, but not the constitutive presence, of the defense response such as high contents of H_2_O_2_ and callose deposition indicates that plants have some mechanisms to suppress inappropriate immunity and keep balances between normal growth and defense responses. It is reasonable to hypothesize that plants must make corresponding changes at the transcriptional and physiological level to deal with the stress from the interfamily transferred NLRs.

Conditional expression of the autoactive NLRs is a promising method to reduce the fitness costs. The TBF1-cassette, a pathogen responsive translational control system, has been successfully used to conditionally regulate the protein level of NPR1, conferring broad-spectrum disease resistance but also avoiding fitness costs (Xu et al., 2017). The transcription activator-like (TAL) effectors can specially bind to the promoter sequences of their target genes. Engineering promoters to trap pathogen TAL effectors from pathogens can specially activate resistance genes at the infection sites (Zeng et al., 2015). Additionally, the global transcription analysis of the plants inoculated with target pathogens can find some pathogen-induced genes. The promoters from these genes can be used to restrict the expression of the autoactive NLRs at infection sites.

## Author Contributions

ZL and ZG designed the study and wrote the manuscript. ZL carried out most experiments and analyzed data. JH, ZW, SZ, and XW contributed to obtain the transgenic plans. FM and ZZ contributed to rice blast assay.

## Conflict of Interest Statement

The authors declare that the research was conducted in the absence of any commercial or financial relationships that could be construed as a potential conflict of interest.

## Supplementary Material

FIGURE S1 Relative expression levels of *AtRPS2* and *AtRPM1*(*D505V*) in the transgenic plants. The expression levels of *AtRPS2* **(A)** and *AtRPM1*(*D505V*) **(B)** in seven independent stable transgenic lines were detected by qRT-PCR assay. The *OsActin* gene was used as an internal control. Data are shown as means ± SD (*n* = 3).

FIGURE S2 Disease resistance assays with detached leaves of the *AtRPS2*, *AtRPM1*(*D505V*) transgenic plants and Nipponbare. **(A)** Detached leaves of 4 weeks old plants were lightly wounded with an ear punch, and 8 μL of spore suspension (5 × 10^5^/mL) of *M. oryzae* isolate was added to the wound sites. Pictures were taken at 7 dpi, bars = 2 cm. **(B,C)** Lesion length and fungal growth were measured at 7 dpi. Data are shown as means ± SD (*n* = 20). Asterisks denote significant differences (one way ANOVA, ^∗∗∗^*P* < 0.001). Similar results were obtained in three independent experiments.

FIGURE S3 GO analysis of differentially expressed genes between *AtRPS2* transgenic plants and Nipponbare.

FIGURE S4 GO analysis of differentially expressed genes between *AtRPM1*(*D505V*) transgenic plants and Nipponbare.

TABLE S1 Segregation analysis of T_1_ *AtRPS2* transgenic plants.

TABLE S2 Segregation analysis of T_1_ *AtRPM1*(*D505V*) transgenic plants.

TABLE S3 Differentially expressed genes (DEGs) between Nipponbare and *AtRPS2* transgenic plants.

TABLE S4 Differentially expressed genes (DEGs) between Nipponbare and *AtRPM1*(*D505V*) transgenic plants.

TABLE S5 Co-regulated genes in *AtRPS2* and *AtRPM1*(*D505V*) transgenic plants.

TABLE S6 List of qRT-PCR primers used in this study.
