# Supplementary figures and images for: Overexpression of Arabidopsis Nucleotide-Binding and Leucine-Rich Repeat Genes RPS2 and RPM1(D505V) Confers Broad-Spectrum Disease Resistance in Rice
Source: Front Plant Sci. 2019 Apr 5;10:417. doi: 10.3389/fpls.2019.00417 (PMC6459959; doi:10.3389/fpls.2019.00417)

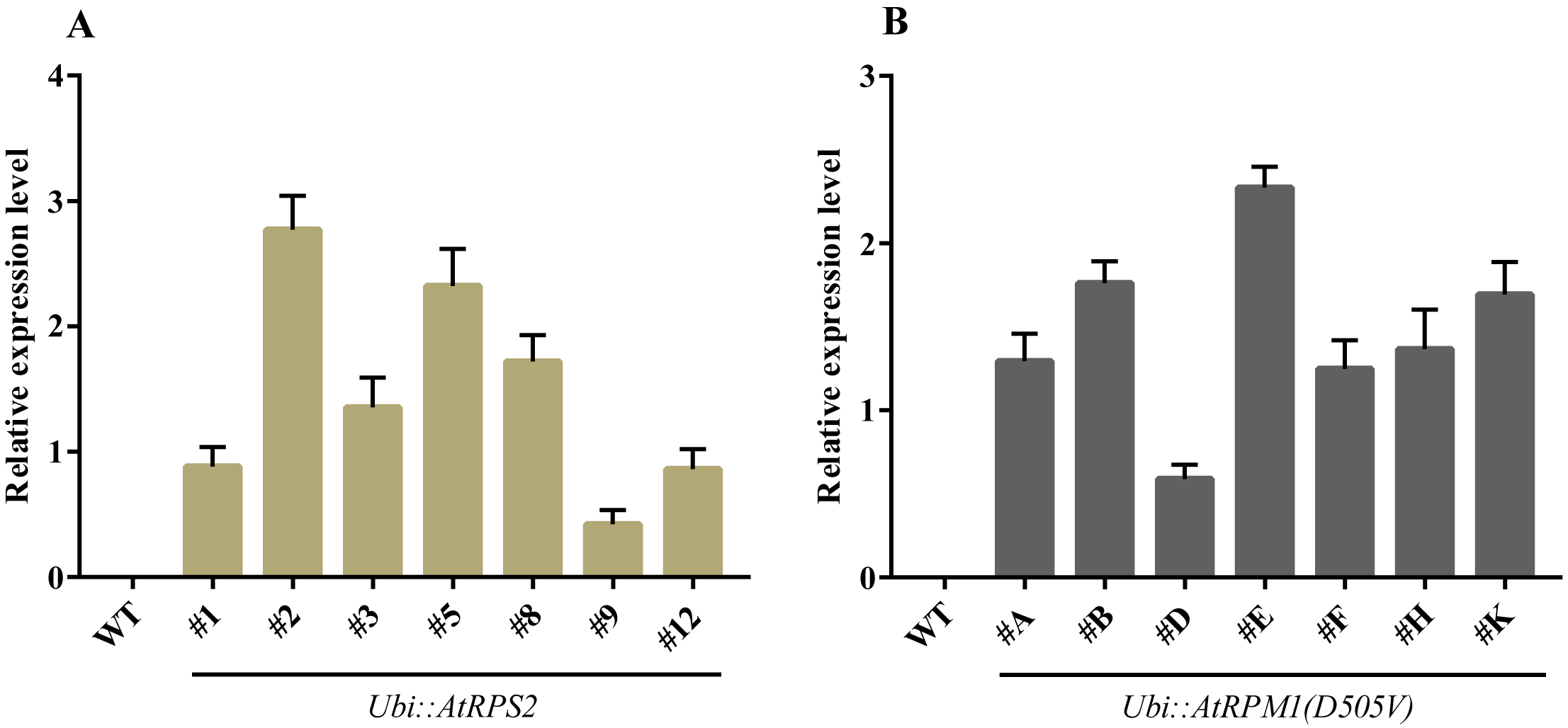

Supplement: FIGURE S1 — Relative expression levels of AtRPS2 and AtRPM1(D505V) in the transgenic plants. The expression levels of AtRPS2 (A) and AtRPM1(D505V) (B) in seven independent stable transgenic lines were detected by qRT-PCR assay. The OsActin gene was used as an internal control. Data are shown as means ± SD (n = 3). [file Image_1.TIF]

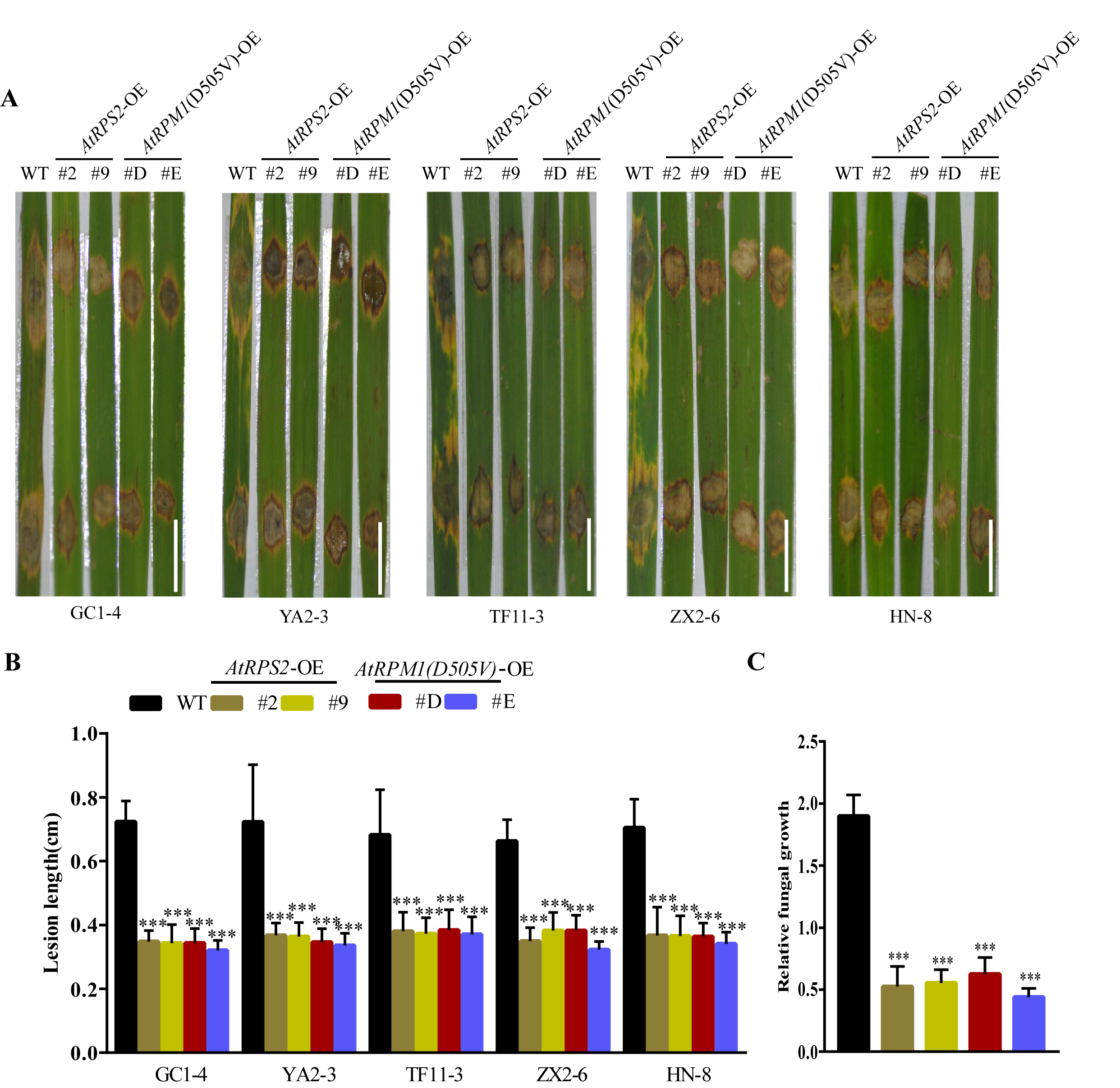

Supplement: FIGURE S2 — Disease resistance assays with detached leaves of the AtRPS2, AtRPM1(D505V) transgenic plants and Nipponbare. (A) Detached leaves of 4 weeks old plants were lightly wounded with an ear punch, and 8 μL of spore suspension (5 × 10^5^/mL) of M. oryzae isolate was added to the wound sites. Pictures were taken at 7 dpi, bars = 2 cm. (B,C) Lesion length and fungal growth were measured at 7 dpi. Data are shown as means ± SD (n = 20). Asterisks denote significant differences (one way ANOVA, ∗∗∗P < 0.001). Similar results were obtained in three independent experiments. [file Image_2.TIF]

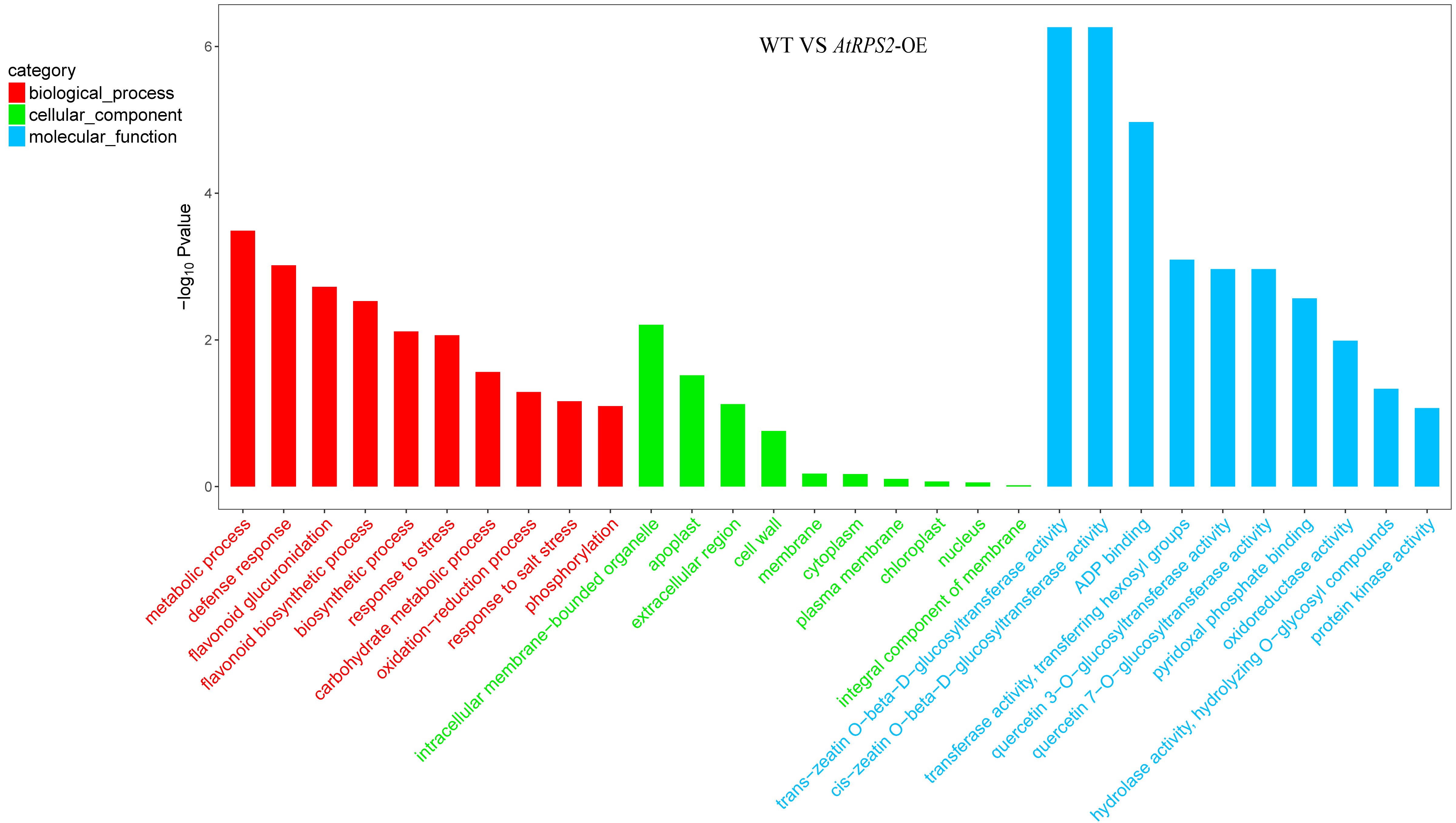

Supplement: FIGURE S3 — GO analysis of differentially expressed genes between AtRPS2 transgenic plants and Nipponbare. [file Image_3.JPEG]

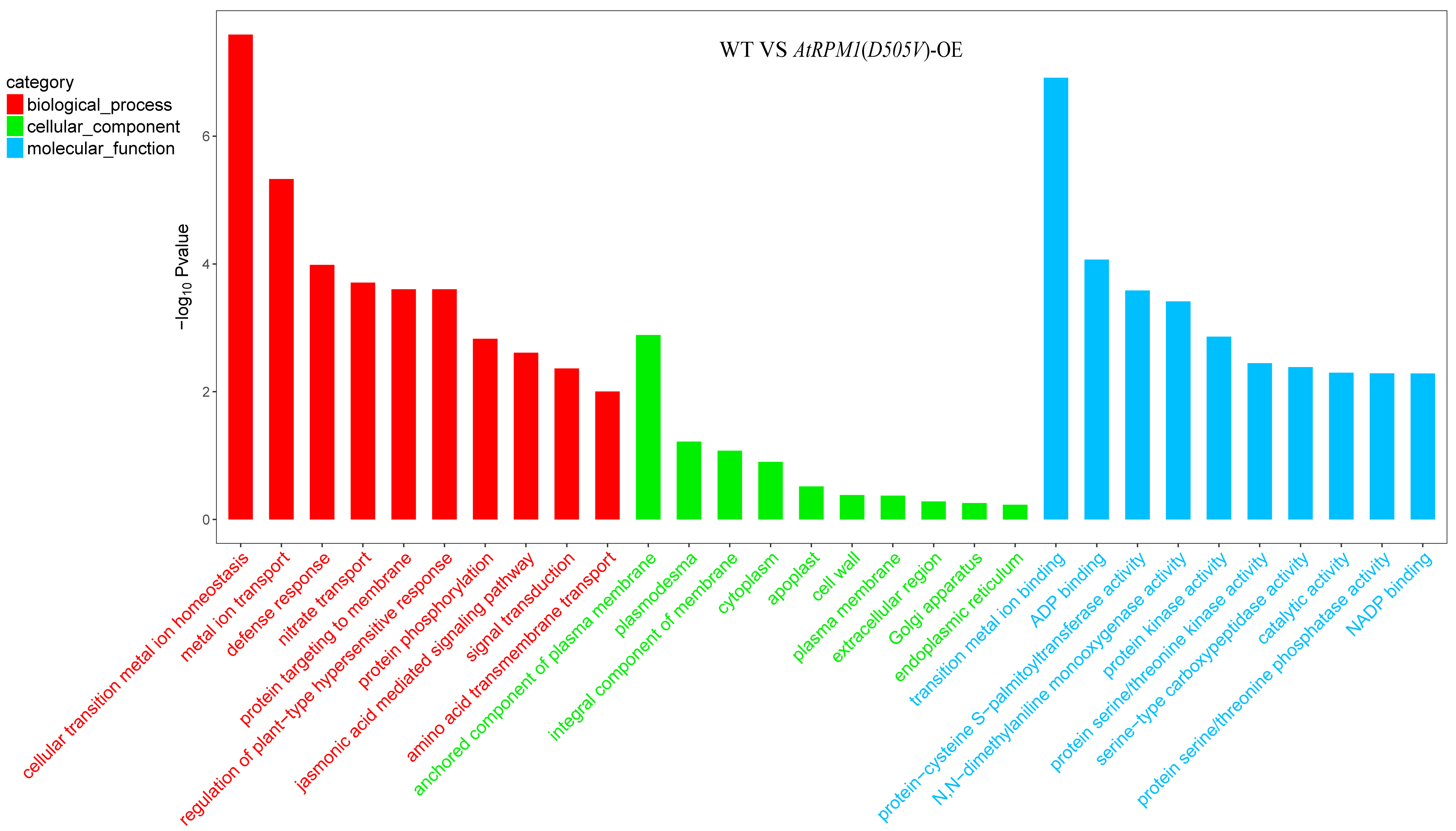

Supplement: FIGURE S4 — GO analysis of differentially expressed genes between AtRPM1(D505V) transgenic plants and Nipponbare. [file Image_4.JPEG]
